# Is outdoor vector control needed for malaria elimination? An individual-based modelling study

**DOI:** 10.1186/s12936-017-1920-y

**Published:** 2017-07-03

**Authors:** Lin Zhu, Günter C. Müller, John M. Marshall, Kristopher L. Arheart, Whitney A. Qualls, WayWay M. Hlaing, Yosef Schlein, Sekou F. Traore, Seydou Doumbia, John C. Beier

**Affiliations:** 1 Department of Public Health Sciences, Miller School of Medicine, University of Miami, Miami, FL USA; 2 Department of Microbiology and Molecular Genetics, IMRIC, Kuvin Centre for the Study of Infectious and Tropical Diseases, Faculty of Medicine, Hebrew University, Jerusalem, Israel; 3 Divisions of Biostatistics and Epidemiology, School of Public Health, University of California, Berkeley, CA USA; 4 Zoonosis Control Branch, Texas Department of State Health Services, Austin, TX USA; 5 Malaria Research and Training Center, Faculty of Medicine, Pharmacy and Odonto-Stomatology, University of Bamako, BP 1805 Bamako, Mali

**Keywords:** Outdoor vector control, Malaria elimination, Residual malaria transmission, *Anopheles gambiae*, Agent-based model, Individual-based model, LLIN, ATSB

## Abstract

**Background:**

Residual malaria transmission has been reported in many areas even with adequate indoor vector control coverage, such as long-lasting insecticidal nets (LLINs). The increased insecticide resistance in *Anopheles* mosquitoes has resulted in reduced efficacy of the widely used indoor tools and has been linked with an increase in outdoor malaria transmission. There are considerations of incorporating outdoor interventions into integrated vector management (IVM) to achieve malaria elimination; however, more information on the combination of tools for effective control is needed to determine their utilization.

**Methods:**

A spatial individual-based model was modified to simulate the environment and malaria transmission activities in a hypothetical, isolated African village setting. LLINs and outdoor attractive toxic sugar bait (ATSB) stations were used as examples of indoor and outdoor interventions, respectively. Different interventions and lengths of efficacy periods were tested. Simulations continued for 420 days, and each simulation scenario was repeated 50 times. Mosquito populations, entomologic inoculation rates (EIRs), probabilities of local mosquito extinction, and proportion of time when the annual EIR was reduced below one were compared between different intervention types and efficacy periods.

**Results:**

In the village setting with clustered houses, the combinational intervention of 50% LLINs plus outdoor ATSBs significantly reduced mosquito population and EIR in short term, increased the probability of local mosquito extinction, and increased the time when annual EIR is less than one per person compared to 50% LLINs alone; outdoor ATSBs alone significantly reduced mosquito population in short term, increased the probability of mosquito extinction, and increased the time when annual EIR is less than one compared to 50% LLINs alone, but there was no significant difference in EIR in short term between 50% LLINs and outdoor ATSBs. In the village setting with dispersed houses, the combinational intervention of 50% LLINs plus outdoor ATSBs significantly reduced mosquito population in short term, increased the probability of mosquito extinction, and increased the time when annual EIR is less than one per person compared to 50% LLINs alone; outdoor ATSBs alone significantly reduced mosquito population in short term, but there were no significant difference in the probability of mosquito extinction and the time when annual EIR is less than one between 50% LLIN and outdoor ATSBs; and there was no significant difference in EIR between all three interventions. A minimum of 2 months of efficacy period is needed to bring out the best possible effect of the vector control tools, and to achieve long-term mosquito reduction, a minimum of 3 months of efficacy period is needed.

**Conclusions:**

The results highlight the value of incorporating outdoor vector control into IVM as a supplement to traditional indoor practices for malaria elimination in Africa, especially in village settings of clustered houses where LLINs alone is far from sufficient.

## Background

Huge progress has been made toward malaria elimination following the scale up of long-lasting insecticidal nets (LLINs) and indoor residual spraying (IRS) [1–5], the two major vector control tools recommended by the World Health Organization (WHO). However, outdoor residual malaria transmission has been consistently reported in many areas where these interventions are in place [6, 7]. The wide use of LLINs and IRS has resulted in increased insecticide resistance in the malaria vector mosquitoes both chemically [8–10] and behaviourally [7, 11–17], reducing the efficacy of these tools and increasing outdoor malaria transmission [18]. Studies have suggested that indoor interventions alone are not sufficient to achieve malaria elimination, especially in places with high malaria transmission [7, 11, 19, 20]. Hence, it is of interest to evaluate the necessity of using outdoor vector control tools to complement the indoor control by LLINs and IRS to achieve malaria elimination.

A major benefit of outdoor vector control is that it can target and kill mosquitoes in their natural outdoor habitats and is not limited only to the vicinity of residential houses. In addition, it can target the vectors displaying more outdoor human biting activity in response to the indoor interventions [7, 11, 12, 18, 21]. There are several outdoor adult mosquito control methods, such as thermal fogging [22, 23], insecticide-treated cattle [24–26], male swarm spraying [27], “push–pull system” (indoor spatial repellent and outdoor traps) [28, 29], and mosquito landing box [30, 31]. There are also outdoor larval source management tools which are recommended by the WHO as supplementary malaria vector control methods [32, 33], but the feasibility is limited because of the large number of small and temporary larval habitats of malaria vectors [34]. One of the most promising new tools for outdoor vector control is attractive toxic sugar baits (ATSBs) [35, 36], which target the sugar-feeding behaviour of mosquitoes [37] and can be used outdoors either sprayed on vegetation or as bait stations. Several field trials have demonstrated the efficacy of both outdoor and indoor ATSBs against malaria vector mosquitoes [35, 36, 38–40]. An individual-based modelling (IBM) study has estimated and compared the effectiveness of different spatial configurations of ATSB treatment on malaria control [41]. In addition, insecticide resistance will not affect the efficacy of this tool because there are already plenty of oral toxins available from different chemical groups and having different modes of actions, which can be used/exchanged in ATSBs [38].

Both LLINs and ATSBs have limited efficacy periods. Studies in multiple locations have reported that LLINs were not fully used because of discarding [42, 43], giving away [42], or using for purposes other than indoor vector control [42]. In addition, the efficacy period of LLIN products varies, and they gradually lose efficacy due to reduced insecticidal activity, holes, damage, and improper use [42–46]. Similar for ATSBs, baits sprayed on vegetation may be washed out by rain, and although bait stations can resist rains, they may lose efficacy due to damage or expiration. Hence, predicting the recovery pattern of vector populations and malaria transmission after the efficacy period of these interventions can provide information on the durability of the interventions to guide the tool replacement schedule and further cost-effectiveness evaluations.

Integrated vector management (IVM) is important to extend the malaria elimination process. IVM suggests evidence-based decision-making and integrated approaches [47]. Considering the option of outdoor vector control, a basic question pertinent to developing IVM programmes is the optimal combination of indoor and outdoor interventions that are required to achieve desired reductions in malaria transmission given financial, technical, and logistical constraints. An IBM study has compared different indoor intervention strategies and suggested outdoor intervention should be added in settings with exophilic vectors; however, outdoor intervention was not evaluated directly in this study [48]. A mathematical modelling study has found that the use of LLINs in combination with ATSBs can achieve significantly better control of anopheline mosquitoes than LLINs combined with IRS, and the ATSB–LLIN combination was relatively effective against outdoor-biting *Anopheles arabiensis* mosquitoes [49]. However, the ATSB intervention was not specified as indoor or outdoor due to lack of a spatial component in the model.

Community trials are appropriate for these evaluations. However, it can be difficult to identify villages as comparable sites in both demographics and environmental factors. In addition, a randomized controlled trial (RCT) is very time and resource-consuming. To avoid ethical consequences, more complicated designs such as stepwise design may be needed. Therefore, a spatial IBM that simulates the life cycle of *Anopheles gambiae* mosquitoes and their interactions with the environment, humans, and the vector control interventions may be the most pragmatic way to initially predict the consequences of combining outdoor and indoor interventions to guide further empirical studies. The objectives of this study are to: (1) compare the immediate (during efficacy period) and long-term (after losing efficacy) reductions of malaria vectors and entomological inoculation rate (EIR) between indoor and outdoor interventions to evaluate the necessity of incorporating outdoor vector control into IVM programme; and (2) estimate the effect of efficacy periods to guide schedule of maintenance and replacement of the intervention tools.

## Methods

### Model design

For this study, a previous IBM that simulated the interactions between *An. gambiae* mosquitoes and humans, environment, and ATSBs was updated with the features of LLINs. The details of the basic model design were introduced in two previous studies, one evaluating the impact of environmental resources on the survival and biting behaviour of *An. gambiae* [50] and the other comparing the effectiveness of different spatial configurations of ATSB treatment [41]. A summary of the model used in this study is as follows:

#### Environment

A hypothetical isolated African village of 600 × 600 m with 25 houses was simulated. To simulate an environment representing the village configuration of clustered houses like those is Mali, the 25 houses were randomly located in the 100 × 100 m center of the simulated environment; to simulate the other type of environment which has dispersed settlement of houses, the 25 houses were randomly located in the whole simulated environment. Fifty natural sugar sources, 50 outdoor resting sites, and 50 larval habitats were randomly placed in the whole village to simulate a “resource rich” environment.

#### Human

A hundred humans were simulated, and they walked randomly outdoors from 7:00 to 19:59, went back home and walked inside their houses from 20:00 to 22:59, after 23:00 they went to bed and were protected by LLINs if covered.

#### Female *Anopheles gambiae*

Female *An. gambiae* were active during night (19:00 to 05:00). Each mosquito had its physiological status recorded in the model: age, energy level, need for blood, and gravid status. In each step, each mosquito performed different behaviours: fly, sugar feed, blood feed, oviposit, or rest, depending on the environmental factors and their own physiological status. The maximum lifespan was 30 days [51–53]. The extrinsic incubation period was 10 days [54, 55]. The mosquitoes were assumed to have a 20% chance of being infected by biting a human once [56–63], natural history of malaria in human was not considered here because of the complexity and uncertainty of malaria immunity [64]. More details of the density-dependent development of mosquito aquatic stages were described in a previous study [41].

#### Intervention

Long-lasting insecticidal nets were used as the example of the indoor intervention since LLINs are the WHO-recommended and commonly used indoor tool. When a LLIN was efficacious, it protected a human sleeping inside it from mosquito bites and killed mosquitoes that contacted with it; no insecticide resistance was simulated in the model because limited data is available on it, additional assumptions could lead to greater uncertainty. Beyond the efficacy period, both physical and chemical protection was removed to mimic holes on the nets and the loss of insecticide efficacy. ATSB is considered one of the best outdoor tools available currently, and so bait stations were placed in 7 × 7 grid configuration [41] over the whole site as the example of the outdoor intervention. The bait stations attracted and killed sugar-feeding mosquitoes. The efficacy for both LLINs and ATSBs were simulated to be binomial (on/off) instead of gradual degrading because data is limited on the degrading pattern of the two tools, and physical damage could be common in both tools, which is consistent with the binomial pattern. In addition, the efficacy periods were increased by month, increasing the resolution to day or second may not have significant impact on the comparisons between different interventions. Three interventions were simulated: 50% LLIN coverage, ATSBs alone, and 50% LLIN coverage plus ATSBs. The three interventions were introduced in different simulations at the beginning of day 1 (the initial equilibration of mosquito population), and efficacy periods (time when interventions remain efficacious) of 30, 60, 90, 120, 150, and 180 days were simulated. Two controls for each of the three interventions were used: one was the negative control, in which no intervention was applied; the other was the positive control, in which continuous efficacy period until the end of the simulation (day 420) was simulated. Each simulation scenario was repeated 50 times.

### Data analysis

Four measures were calculated and analysed to examine the effects of different interventions and efficacy periods. To describe the best possible effects while the interventions were efficacious, means of mosquito population sizes and annual EIRs at the last day of the efficacy periods (e.g. day 60 for ATSB intervention with an efficacy period of 60 days, day 90 for 50% LLIN coverage with an efficacy period of 90 days) were compared between different interventions and efficacy periods. To control for the over dispersion and fit the data distribution, negative binomial regression models with a square root transformation of the outcomes were selected. Intervention type, efficacy period, and their interaction were included in the model. The annual EIRs were derived as daily EIR times 365 days, daily EIRs were calculated as the total number of infectious bites per human per day. A value of 0.0001 was added to EIRs to eliminate cells with zero variance and enable model fitting. To describe the long-term effects after the interventions lost efficacy, the probabilities of mosquito population extinction at the end of simulation (day 420) were compared between different interventions and efficacy periods using a logistic regression model. Intervention type, efficacy period, and their interaction were included in the model. Interventions that had none of the 50 trials ending up with extinction were excluded from the analysis. To describe the overall effects, the total number of days while EIR was reduced to the level below one per person per year for each intervention and efficacy period were compared using a negative binomial regression model.

## Results

The environment simulated in this model represented a malaria endemic village: in the 600 × 600 m area, female *An. gambiae* mosquito populations equilibrated at around 400 without any intervention. Annual EIR equilibrated at around 200 infectious bites per person per year.

**Fig. 1:**
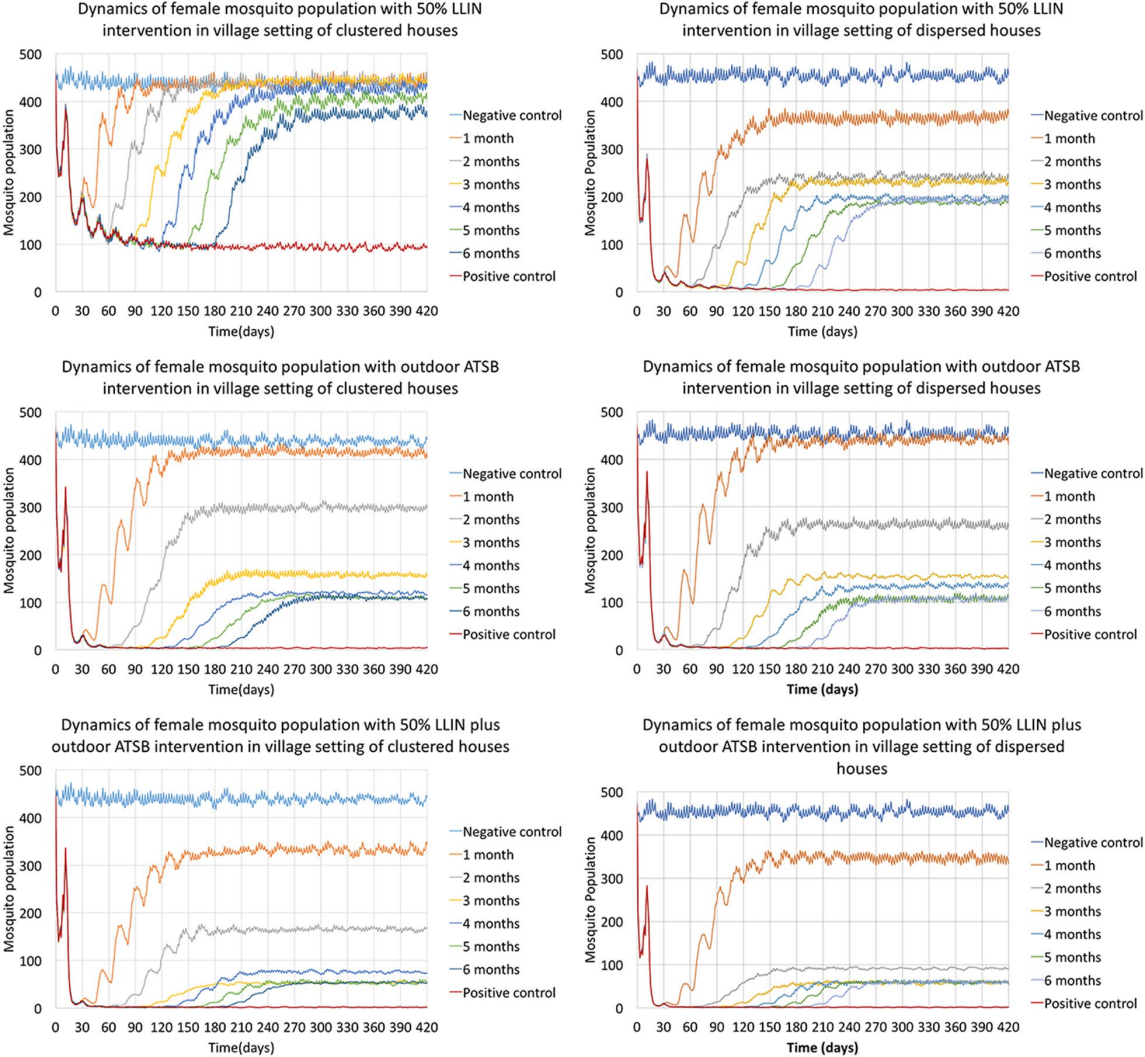
Dynamics of female mosquito population with different intervention types and efficacy periods in clustered and dispersed village settings. The *two columns of figures* show the dynamics of female mosquito populations in village setting of clustered houses (*on the left*) and dispersed houses (*on the right*). The *first row of figures* shows results of 50% LLIN intervention, the *second row* shows results of outdoor ATSB intervention, and the *third row* shows results of 50% LLIN plus ATSB intervention. In all *six figures*, the y-axis represents the mean number of female *An. gambiae* mosquitoes at each day, and the x-axis shows the time in days. Day 0 is the day that the intervention was applied. The *lines in different colors* represent different simulated efficacy periods for each intervention

Figure 1 shows the dynamics of female mosquito population with different intervention types and efficacy periods in the two types of village configurations. Mosquito populations dropped sharply in the first 30 days following the treatments and then decreased slowly toward equilibration in the next 30 days. Mosquito populations began to reestablish immediately after the efficacy period of the interventions. However, with different types of interventions and efficacy periods, the re-equilibrated mosquito populations averaged at different levels. The mosquito populations were the means (averages) of 50 repeated simulations. In a proportion of the 50 repetitions, mosquito populations were reduced to very low levels and reestablished to the negative control level. In the other repetitions, mosquito populations were annihilated, so smaller means of re-equilibrated population sizes represent lower chances of recovery to a negative control level. Interventions of or with outdoor ATSB treatment led to better mosquito control results, especially in village setting of clustered houses. The detailed comparison of immediate and long-term effects of different interventions and efficacy periods in the two village configurations are presented in Tables 1, 2 and 3.

**Table 1:** Average population sizes and EIRs at the last day of efficacy periods for different intervention types and efficacy periods

Village configurations	Outcomes	Intervention	Efficacy period (months)													
			1		2		3		4		5		6		14*	
			Mean	% reduction	Mean	% reduction	Mean	% reduction	Mean	% reduction	Mean	% reduction	Mean	% reduction	Mean	% reduction
Centered houses	Population size	Negative control	433.95	0.00	424.01	0.00	438.26	0.00	430.98	0.00	437.42	0.00	422.41	0.00	442.57	0.00
		LLIN 50%	193.14	55.49	99.40	76.56	109.09	75.11	94.98	77.96	98.88	77.39	94.41	77.65	91.70	79.28
		ATSB	27.20	93.73	5.42	98.72	4.01	99.09	4.04	99.06	3.90	99.11	3.19	99.24	4.82	98.91
		LLIN 50% plus ATSB	14.39	96.68	2.05	99.52	1.61	99.63	1.56	99.64	1.53	99.65	2.08	99.51	1.88	99.58
	Annual EIR	Negative control	233.67	0.00	205.42	0.00	209.07	0.00	213.45	0.00	195.71	0.00	212.21	0.00	188.63	0.00
		LLIN 50%	3.50	98.50	1.39	99.32	2.48	98.81	1.17	99.45	0.80	99.59	1.39	99.34	1.90	98.99
		ATSB	0.44	99.81	0.00	100.00	0.07	99.97	0.00	100.00	0.00	100.00	0.07	99.97	0.00	100.00
		LLIN 50% plus ATSB	0.00	100.00	0.00	100.00	0.00	100.00	0.00	100.00	0.07	99.96	0.00	100.00	0.00	100.00
Dispersed houses	Population size	Negative control	432.91	0.00	452.09	0.00	447.40	0.00	440.69	0.00	444.92	0.00	458.41	0.00	454.09	0.00
		LLIN 50%	35.62	91.77	10.67	97.64	9.24	97.93	6.86	98.44	4.91	98.90	5.89	98.72	4.39	99.03
		ATSB	30.43	92.97	4.92	98.91	4.33	99.03	2.45	99.44	2.92	99.34	3.54	99.23	2.58	99.43
		LLIN 50% plus ATSB	7.47	98.27	1.70	99.62	1.59	99.64	1.69	99.62	1.18	99.73	1.71	99.63	1.55	99.66
	Annual EIR	Negative control	266.74	0.00	250.61	0.00	240.53	0.00	260.32	0.00	245.65	0.00	284.92	0.00	256.38	0.00
		LLIN 50%	0.37	99.86	0.00	100.00	0.00	100.00	0.22	99.92	0.00	100.00	0.00	100.00	0.00	100.00
		ATSB	0.00	100.00	0.07	99.97	0.00	100.00	0.15	99.94	0.00	100.00	0.00	100.00	0.07	99.97
		LLIN 50% plus ATSB	0.00	100.00	0.00	100.00	0.00	100.00	0.07	99.97	0.22	99.91	0.00	100.00	0.00	100.00

* Positive control, which is continuous efficacious intervention until the end

**Table 2:** Proportions (%) of trials ending up with local mosquito extinction for different intervention types and efficacy periods

Village configuration	Intervention	Efficacy period (months)							
		Negative control	1	2	3	4	5	6	Positive control
Clustered houses	LLIN 50%	0	0	0	0	4	6	12	34
	ATSB		12	42	70	78	80	80	80
	LLIN 50% plus ATSB		28	68	90	86	90	90	90
Dispersed houses	LLIN 50%	0	24	54	58	64	66	66	80
	ATSB		4	48	70	74	80	80	84
	LLIN 50% plus ATSB		30	84	90	90	90	90	90

**Table 3:** Proportions (%) of time when EIR was below 1 per person per year for different intervention types and efficacy periods

Village configuration	Intervention	Efficacy period (months)							
		Negative control	1	2	3	4	5	6	Positive control
Clustered houses	LLIN 50%	13.68	20.31	26.05	31.55	38.95	46.66	52.78	86.10
	ATSB		25.59	53.31	76.68	83.74	86.32	87.62	97.96
	LLIN 50% plus ATSB		43.34	74.27	91.73	89.99	93.16	93.67	98.77
Dispersed houses	LLIN 50%	4.35	36.16	62.57	67.74	74.26	78.28	80.59	97.98
	ATSB		20.68	58.78	76.95	81.69	86.49	87.76	97.76
	LLIN 50% plus ATSB		42.31	86.34	91.63	92.20	93.03	93.60	99.00

Table 1 shows the means of mosquito population sizes and annual EIRs at the last day of the efficacy periods for different intervention types and efficacy periods. In the village setting with clustered houses, female population size was reduced from over 400 in negative control to a range of 2–193, and annual EIR was reduced from over 200 to a range of 0–4 with different intervention types and efficacy periods. The results of the negative binomial regression model show that all three interventions reduced mosquito population and EIR to significantly lower levels than negative control (Ps < 0.0001), outdoor ATSB intervention reduced mosquito population and EIR to significantly lower levels than 50% LLIN intervention (P < 0.0001), the intervention of 50% LLINs plus outdoor ATSBs reduced mosquito population to significantly lower level than the intervention of ATSBs alone (P < 0.0001), however, EIR was not significantly different between the combinational intervention and outdoor ATSB alone (P = 0.3178). Interventions with efficacy period of 2 months or longer reduced mosquito population to significantly lower levels than efficacy period of 1 month (Ps < 0.0001), and there was no significant difference in EIR between different efficacy periods (Ps > 0.05).

In the village setting with dispersed houses, female population size was reduced from over 400 in negative control to a range of 1–36, and annual EIR was reduced from over 200 to a range of 0–0.4 with different intervention types and efficacy periods. All the three interventions reduced population size and EIR to significantly lower levels than negative control (P < 0.0001). Although the differences of population size between each intervention type were still statistically significant (Ps < 0.0001), the absolute differences between 50% LLIN and outdoor ATSB were much smaller than those in the village setting with clustered houses. There was no significant difference in EIR between the three interventions (P > 0.05). In addition, there was no significant difference in EIR between different efficacy periods (P > 0.05).

Table 2 shows the probabilities (proportions of trials) of mosquito extinction for each intervention type and efficacy period after the interventions lost efficacy. In the village setting of clustered houses, the probability of mosquito extinction was increased from 0 to a range of 0–90% by the three interventions. Outdoor ATSB intervention increased the probability to significantly higher levels than 50% LLIN intervention (P < 0.0001), and the intervention of 50% LLINs plus outdoor ATSBs increased the probability to significantly higher level than the intervention of outdoor ATSBs alone (P < 0.0001). Longer efficacy periods significantly increased the probability of mosquito extinction (Ps < 0.0001), except that there was no significant difference between efficacy periods of 3–6 months (Ps > 0.05).

In the village setting of dispersed houses, the probability of mosquito extinction was increased from 0 to a range of 4–90% by the three interventions. The intervention of 50% LLINs plus outdoor ATSBs increased the probability to significantly higher level than the other two interventions (Ps < 0.0001), but there was no significant difference between the intervention of 50% LLINs and outdoor ATSB alone (P = 0.2286), except that with efficacy period of 1 month, 50% LLINs intervention led to a significant higher probability than outdoor ATSB intervention (P = 0.0108). The comparison between different efficacy periods was similar with that in the village setting of clustered houses: longer efficacy periods significantly increased the probability of mosquito extinction (Ps < 0.0001), except that there was no significant difference between efficacy periods of 3–6 months (Ps > 0.05).

Table 3 shows the proportion of time that EIR was reduced to below one per person per year for different intervention types and efficacy periods. In the village setting of clustered houses, the proportion of time that EIR was less than one was increased from 13.68% to a range of 20.31–98.77% by the three interventions. Outdoor ATSB intervention increased the proportion of time to significantly higher levels than 50% LLIN intervention (P < 0.0001), and the intervention of 50% LLINs plus outdoor ATSBs increased the probability to significantly higher level than the intervention of outdoor ATSBs alone (P = 0.0002). Longer efficacy periods significantly increased the proportion of time most of times (Ps < 0.05), except that there was no significant difference between efficacy periods of 3 and 4 months (P = 0.2666), 4 and 5 months (P = 0.3015), 4 and 6 months (P = 0.0940), and 5 and 6 months (P = 0.5207).

In the village setting of dispersed houses, the proportion of time that EIR was less than one was increased from 4.35% to a range of 20.68–99.00% by the three interventions. The intervention of 50% LLINs plus outdoor ATSBs increased the probability to significantly higher level than the other two interventions (Ps < 0.0001), but there was no significant difference between the intervention of 50% LLINs and outdoor ATSB alone (P = 0.6493), except that with efficacy period of 1 month, 50% LLINs intervention led to a significant higher proportion than outdoor ATSB intervention (P < 0.0001). Longer efficacy periods significantly increased the proportion of time most of times (Ps < 0.05), except that there was no significant difference between efficacy periods of 3–6 months (Ps > 0.05).

## Discussion

In this study, the potential benefits of incorporating outdoor vector control into the widely used indoor vector control strategy (LLINs) in a malaria endemic village were evaluated by simulating three types of indoor and outdoor control interventions with different efficacy periods. According to WHO recommendations and current research findings, LLINs with 50% coverage were used as the example of indoor mosquito control; a pattern of ATSB stations placed in a 7 × 7 grid configuration over the whole area was used as the example of outdoor mosquito control. Incorporating outdoor ATSB intervention significantly improved both immediate and long-term effects of mosquito and malaria control, especially in the village setting with clustered houses.

The mosquito population size and EIR at the last day of efficacy periods is the best possible effect of an intervention with a certain efficacy period, which represents the immediate effect. The results show that all three types of interventions are effective at reducing mosquito population and EIR, however, to achieve a better mosquito control result, outdoor ATSBs alone or the combination of 50% LLINs and outdoor ATSBs is suggested, especially in village setting of clustered houses where even outdoor ATSBs alone achieved substantially lower population size than 50% LLINs. The difference in EIR between different interventions was smaller, thus to only achieve EIR reduction, if resources are limited, outdoor ATSBs alone in village setting of clustered houses is acceptable, and either 50% LLINs or outdoor ATSBs alone in village setting of dispersed houses is acceptable. In addition, for any interventions, a minimum of 2 months of efficacy period is needed to bring out the best possible effectiveness. This provides guidance for schedule of tool maintenance and education for the human compliance.

The probability of local mosquito extinction after the interventions lose efficacy represents the long-term effect. The explanation for the long-term mosquito reduction may be that a good combination of interventions can rapidly knock down the mosquito populations to very low levels; and after a certain time, as the existing eggs develop to adults and are killed and as overall reproduction decreases, the population can be locally eliminated. In both village settings, the combinational intervention of 50% LLIN and outdoor ATSBs was suggested to achieve the best long-term mosquito control results. Even with very limited resources, intervention of 50% LLINs alone is not suggested in village setting of clustered houses. In addition, a minimum of 3 months of efficacy period is needed to achieve the best long-term mosquito reduction.

There is a study showing that malaria elimination can be achieved when annual EIR is below one [65]. The results of the proportion of time when annual EIR is less than one represents the overall protection. In both village settings, incorporating outdoor ATSBs into the LLIN intervention with an efficacy period of 3 months or longer achieved the same level of protection as the other two interventions with continuous efficacy until the end of simulation. This suggests that an investment of the combinational intervention short-term may save the work of long-time maintenance of the interventions.

The underlying mechanism for the increase in effectiveness by the incorporation of the outdoor ATSB intervention is that the outdoor ATSBs not only killed mosquitoes that entered houses, but also killed those searching for sugar meals outdoors. LLINs only target the mosquitoes searching for a blood meal indoors, and these mosquitoes constitute a proportion of the mosquito population since females would blood feed mainly once per gonotrophic cycle (3 days in average) [66–68]. Whereas outdoor ATSBs target the mosquitoes searching for sugar meals, which constitute a larger proportion of the mosquito population since they require at least one sugar meal per night for energy and more activity would increase the needs for sugar meals [37, 69]. In addition, ATSBs can well target male *An. gambiae* mosquitoes because males solely feed on sugar for energy and they require at least two sugar meals per night [69]. Male mosquitoes were simulated in ten repetitions and followed similar population dynamic patterns as females, so they were not included in the results. However, as outdoor ATSBs killed more male mosquitoes, it would reduce the mating opportunities for females, which would either lead to lower mosquito reproduction or additional flights for females and hence increased mortality. Successful mating was assumed in the model, if mating opportunity was taken into account, outdoor ATSB intervention may have even better vector control results. In the current model, a sugar-rich village was simulated. Whereas in villages that lack natural sugar resources, or during dry seasons, the outdoor ATSB intervention could achieve even better results [41].

A LLIN coverage of 50% was used in this study, this is due to the fact that according to the estimation by WHO, the average bed net coverage increased from less than 2% in 2000 to an estimated 55% in 2015 [70]. Except for the considerations of incorporating outdoor vector control, there are also considerations of scaling up LLIN coverage for malaria elimination. However, most studies evaluating the effectiveness of scaling up LLIN coverage investigated the effect where the coverage rate increased from very low to intermediate/high level and showed significant reduction of malaria transmission [71–74], but there is no empirical evidence showing that further increasing the coverage can reduce malaria transmission: only one study evaluated locations with this magnitude of increase, and the results showed that asexual parasite prevalence decreased significantly, but gametocyte prevalence did not [75]. In addition, results from another RCT showed that universal LLIN coverage did not reduce malaria transmission than targeted LLIN coverage for pregnant women and children under six [76]. These are consistent with the results of 100% LLIN intervention in this study, which are not reported here. The results of the simulations show that there was no significant difference between 100% coverage and 50% coverage. Another explanation for this phenomenon may be that no insecticide resistance was simulated for LLIN. However, with 50% LLIN coverage, if behavioural resistance in mosquitoes was simulated, the mosquitoes could avoid LLINs and select those unprotected human for blood-feeding. Since those unprotected humans could then compensate the reduced bites for those protected under LLINs, the EIR could be higher in 50% LLINs in reality, and thus the difference between 50 and 100% coverages could be greater. Because of the simplification of not including insecticide resistance in this model, the relevant results can be inaccurate and are not included. Further modelling studies with these details and empirical studies are needed for the confirmation of the effectiveness of scaling up LLIN coverage.

One limitation of the study was that the simulated village was isolated/closed to influx of mosquitoes from the outside. Thus, after the mosquito population was knocked down, the effect could be sustained even after the loss of interventions efficacy. However, previous studies have shown that malaria can be locally eliminated when vector density is below a certain threshold [77, 78]. If the influx of mosquitoes is below a certain level and not able to reestablish the population, the effect of control may still be sustained. In addition, anopheline mosquitoes have a maximum dispersal range from several 100 m to almost 3000 m [79]. With limited human migration between villages, if the villages are separated by distances exceeding the maximum dispersal range, especially in arid environments, or if several villages are treated at the same time in a big area exceeding the maximum dispersal range, then “isolation” can be achieved and local extinction may be sustained.

In this study, ATSB stations placed outdoor in 7 × 7 grid configuration over the whole area was used to represent outdoor adult mosquito control in general. ATSB attracts sugar-seeking mosquitoes from a distance and are not limited by constraints of insecticide resistance; in fact, ATSB is being considered as a new tool for breaking insecticide resistance. Generalization from the study results from this study to other available outdoor vector control tools should be careful because of these advantages of ATSB tool. No insecticide resistance was simulated for the indoor LLIN treatments, and no damage was simulated for the outdoor ATSB treatments during its efficacy period. However, in reality, these perfect conditions are never achieved. Therefore, this study may have over-estimated the effectiveness of both tool. Nevertheless, even with the simplifications in this modelling study, it provides insights of how expanding mosquito control from inside houses to a larger scale outdoors can benefit overall vector and malaria control.

## Conclusions

In summary, incorporating outdoor vector control interventions such as ATSBs into IVM strategies is suggested for malaria elimination, especially in village setting of clustered houses where indoor LLINs alone are far from sufficient. With the combination of indoor and outdoor vector control interventions, it is likely that mosquito population will become locally extinct even after the efficacy period of the interventions in certain conditions, leading to long-term mosquito control and EIR reduction. Further confirmation from empirical studies are recommended to provide additional evidence for updating the malaria elimination policy.

## Abbreviations

LLIN: long-lasting insecticidal net
ATSB: attractive toxic sugar bait
IVM: integrated vector management
EIR: entomological inoculation rate
WHO: World Health Organization
RCT: randomized controlled trial
